# Functional Properties of Pea (*Pisum sativum*, L.) Protein Isolates Modified with Chymosin

**DOI:** 10.3390/ijms12128372

**Published:** 2011-11-29

**Authors:** Miroljub Barać, Slavica Čabrilo, Mirjana Pešić, Slađana Stanojević, Milica Pavlićević, Ognjen Maćej, Nikola Ristić

**Affiliations:** 1 Faculty of Agriculture, University of Belgrade, Nemanjina 6, Belgrade-Zemun 11000, Serbia; E-Mails: mpesic@agrif.bg.ac.rs (M.B.); sladjas@agrif.bg.ac.rs (S.S.); mpavlicevic@agrif.bg.ac.rs (M.P.); macej@agrif.bg.ac.rs (O.M.); nristic@agrif.bg.ac.rs (N.R.); 2 High Technical School of Vocational Studies, Nemanjina 2, Pozarevac 12000, Serbia; E-Mail: slavica.cab@sbb.rs

**Keywords:** partial hydrolysis, chymosin, isolate, functional properties

## Abstract

In this paper, the effects of limited hydrolysis on functional properties, as well as on protein composition of laboratory-prepared pea protein isolates, were investigated. Pea protein isolates were hydrolyzed for either 15, 30 and 60 min with recombined chymosin (Maxiren). The effect of enzymatic action on solubility, emulsifying and foaming properties at different pH values (3.0; 5.0; 7.0 and 8.0) was monitored. Chymosin can be a very useful agent for improvement of functional properties of isolates. Action of this enzyme caused a low degree of hydrolysis (3.9–4.7%), but improved significantly functional properties of pea protein isolates (PPI), especially at lower pH values (3.0–5.0). At these pH values all hydrolysates had better solubility, emulsifying activity and foaming stability, while longer-treated samples (60 min) formed more stable emulsions at higher pH values (7.0, 8.0) than initial isolates. Also, regardless of pH value, all hydrolysates showed improved foaming ability. A moderate positive correlation between solubility and emulsifying activity index (EAI) (0.74) and negative correlation between solubility and foam stability (−0.60) as well as between foam stability (FS) and EAI (−0.77) were observed. Detected enhancement in functional properties was a result of partial hydrolysis of insoluble protein complexes.

## 1. Introduction

For a long time enzymatic hydrolysis has been recognized as relatively simple and a useful method for improving sensory and nutritive values of plant proteins. Limited enzymatic hydrolysis is also the safest way to obtain desirable functional properties such as gelling, foaming and emulsifying of plant based protein products Also, the latest research [1–3] has shown that proteases-treated plant proteins possessed good antioxidant activities.

Proteins of different plant sources such as soy [4–7], pea bean [1,2,8,9], rice [10], peanuts [11,12] cowpea [13] were the object of limited enzymatic hydrolysis. Different enzymes, including trypsin [14,15] papain [16–18], pepsin [4,19,20] and several commercial proteases with different activity [11,21–23], were used. It is well known that the effect of enzymatic hydrolysis is dependent on numerous factors such as type of enzyme [24,25] and treatment conditions (enzyme-substrate ratio, reaction temperature, time of enzyme action, characteristics of substrate) [21]. Thus, in order to achieve optimal results, hydrolysis must be carried out under strictly defined and controlled conditions.

Generally, plant protein hydrolysates can be classified into two groups, partially hydrolyzed (degree of hydrolysis (DH) < 10%) and intensively hydrolyzed (DH > 10%) [26]. Hydrolysis to about DH 10% contributed to an improved solubility, emulsifying, foaming and other functional properties. In contrast, intensively hydrolyzed plant proteins were characterized by poor functional properties, but good nutritive values.

Most research has focused on limited hydrolysis of soy protein products, while less attention was devoted to pea protein products. Research by Aluko *et al.* [27], Maninder *et al.* [28], and Barac *et al.* [29] showed that pea (*Pisum sativum*, L.) protein products could be very effective functional additives and a good substitute for soy proteins. Dominant pea proteins are globulins, which are usually classified, according to the sedimentation coefficient, into two fractions, 7S and 11S. The dominant components of these fractions were legumin (11S protein), vicilin and convicilin (7S proteins). Properties of these proteins are well documented [30–34], so significant differences in amino acid composition, and structure and, consequently, different functionality of these proteins are already known [35].

Chymosin belongs to A1-family of protease and is characterized with broad specificity similar to that of pepsin A. It is primarily used for the enzymatic phase of coagulation of milk proteins. It is widely produced as recombined enzyme from genetically modified yeasts and has many commercial names, e.g. Maxiren. Due to low proteolytic activity and low cost, chymosin might be suitable to improve the functional properties of plant protein. The possibility of its application in this sense is currently under debate. An exception is research of Sissons and Thurston [36], which examined the influence of chymosin on soy proteins. They showed that this enzyme had no significant proteolytic activity against native form of glycinin. In contrast, Stanojevic *et al.* [37] proved that chymosin hydrolyzed thermally-treated soy proteins. In current literature, there is no data about the effect of particular enzymes on protein composition of pea isolates or on their functional properties. Therefore, the aim of this study was to determine the applicability of chymosin as agents for improvement of solubility, emulsifying and foaming properties of pea protein isolates and to characterize changes of protein composition produced by enzymes. A relatively inexpensive, high quality functional additive could be obtained by the action of this enzyme.

## 2. Results and Discussion

### 2.1. Degree of Hydrolysis

**Figure 1:**
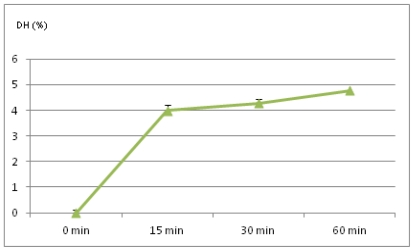
Enzymatic hydrolysis curves of pea protein isolate (PPI) obtained with chymosin.

Enzymatic hydrolysis curves of pea protein isolate (PPI) obtained with chymosin are presented in Figure 1. As could be expected, chymosin had low proteolytic activity against pea protein isolate. Depending on the time of proteolysis, DH values were 3.9–4.7%, and were similar to the results obtained with trypsin by Karamać *et al.* [38]. DH values chymosin-modified isolates were statistically significant at *p <* 0.05. According to these values, modified isolates could be classified as low-modified products that should have better functional properties than the initial isolates.

Hydrolysis curves were quite similar to those produced by other enzymes. Namely, rapid hydrolysis was observed at the beginning of the reaction (first 15 min). Further treatment caused less intensive hydrolysis. Such behavior can be considered as a common characteristic of enzymatic reactions, and has been reported for several other commercial proteases acting on proteins from different legumes [21,24].

### 2.2. PAGE and SDS-PAGE

**Figure 2:**
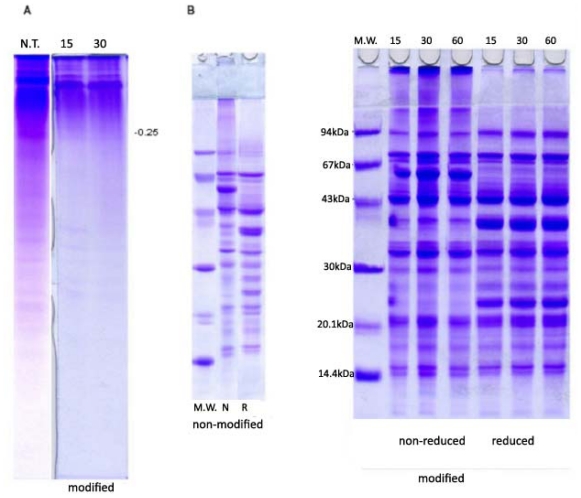
PAGE (**A**) and SDS-PAGE (**B**) analysis of non-modified and modified isolates.

Protein composition of PPI and the changes produced with chymosin were monitored by PAGE and SDS-PAGE. Results are presented in Figure 2. By methods applied in this work, proteins from non-modified isolates were separated into large numbers of fractions. Similar to other leguminoseas, under non-dissociative and non-reducing conditions, predominant pea proteins were showing a tendency toward re-association. Thus, bands at PAGE patterns of non-modified isolate represented their soluble complexes. Two zones—one zone of low electrophoretic mobility (Em < 0.25) and one of higher Em (0.25–1.0)—were visible on all native PAGE profiles. Fractions of high molecular mass and low mobility were dominant, as evidenced by PAGE-profiles. SDS-PAGE under reducing and non-reducing conditions separated total pea bean proteins into multiple components with molecular weight (MW) ranging from 104.8 kDa to 9.8 kDa, which originated mainly from vicilin and legumin. The SDS-PAGE profiles of Tris-extracts contained three major (47.3, 35.0, 28.7 kDa) and three minor (37.0, 33.3, 31.8 kDa) subunits of vicilin, as well as two subunits of convicilin (MW 77.9 kDa, 72.4 kDa). Legumin was identified with four bands of acidic (MW 40.89 kDa) and basic (22.3, 23.1 kDa) subunits. Under reducing condition, three minor bands of monomeric (non-reduced) form of legumin (MW 63.5 kDa) were detected. Non-reducing conditions promoted the re-association of legumin subunits into monomeric form that was registered as intensive band with the same MW. Also, the minor bands of 92.7 kDa and 11.5 kDa were identified as lypoxigenase (Lox) and protease inhibitor (PI), respectively. The molecular weight of identified subunits and polypeptides calculated based on the Em value was consistent with the previous work of several authors [39,40].

Both types of profiles of modified isolates, native and SDS-, reflected low level of hydrolysis produced with chymosin and were qualitatively similar to profiles of non-modified isolate. This data was in agreement with the results of DH analysis, although some differences could be observed on both native and SDS profiles. After 15 min of hydrolysis, bands with Em in the range of 0–0.25 visible on PAGE gel were reduced, whereas diffuse zone bellow these Em values disappeared completely. Further hydrolysis (60 min) induced almost complete breakdown of minor fractions in the Em range 0.5–0.8. From SDS-PAGE profiles under non-reducing conditions difference between non-modified and modified samples was observed in the range of high molecular weight (>90 kDa). With modified samples, high molecular weight fractions could be seen on the stacking gel as well as on the top of the resolving gel. Such fractions were not registered on the stacking gel of non-modified isolates. Under reducing conditions (in the presence of 2-mercaptoethanol) these fractions almost completely disappeared. This indicated that most of these aggregates were formed through disulfide interactions. Pea proteins in general had low content of sulfur amino acids. The exception was legumin. Above infers that most of these aggregates probably resulted from interactions between partly degraded subunits of legumin. Part of these products under non-reducing and especially under reducing conditions migrated as band with MW of 94 kDa and masked a less intensive band of lypoxigenase.

The SDS-PAGE profiles (Figure 2B) under reducing and non-reducing conditions were characterized with intensive bands of legumin and vicilin. Such results led to two conclusions. First, these proteins were stable against chymosin action, which was in agreement with the results of Sissons and Thurston [36] based on pure soy proteins. Secondly, activity of enzyme was primarily directed to the degradation of non-soluble aggregates that were formed during the preparation of isolate. Due to degradation, significant changes of vicilin to legumin ratio, as well as sum of convicilin and vicilin to legumin ratio were registered. Depending on duration of hydrolysis V/L ratio increased from 1.15 to 1.32 (30 min)–1.42 (15 min) whereas (V+C/L) ratio increased from 1.46 to 1.74–1.92 (Table 1). According to results of densitometric analysis, such an increase was primarily a consequence of the lower concentration of legumin subunits.

### 2.3. Solubility

**Figure 3:**
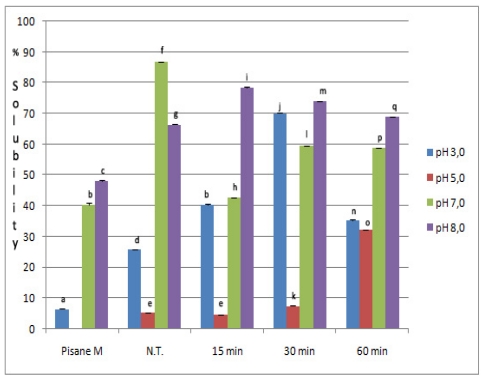
Solubility of commercial, non-treated (N.T.) and modified pea protein isolates at different pH values *. * Bars with the same letter were not different (*p* < 0.05). Means were of triplicate determinations.

Solubility was measured at pH 3.0, 5.0, 7.0 and 8.0, since foams and emulsions were produced at those pH values. Solubility of native, commercial and modified pea protein isolates is presented in Figure 3. In general, chymosin improved solubility of PPI. Except at pH 7.0, modified isolates had better solubility than initial isolate at all pH values. For example, at pH 5.0 samples treated for 60 min had about six times higher solubility. Also, at all pH, solubility of isolates was significantly higher compared to commercial isolate. Yet, obtained values were significantly different (at *p* < 0.05).

Especially good solubility of modified isolates was achieved in the range of low pH values (3–5). Depending on the time of hydrolysis, at pH 3.0, solubility of isolates increased by 40.61–175.95% with a maximum value reached after 30 min (71.14%), while at pH 5.0 this parameter was improved in samples hydrolyzed for 30 and 60 min (7.51%; 34.22%). Similarly, at pH 8.0, solubility reached maximum after 15 min (75.07%), and then slightly decreased. In contrast, at pH 7.0, solubility of chymosin-modified samples was lower than the initial isolates and ranged from 38.27% to 62.61%. These results were consistent with previous reports of Tsoukala *et al.* [8] that had shown trypsin to produce protein hydrolysates with lower solubility at pH values near 7.0 than unmodified ones. Nevertheless, even at this pH value the solubility of the modified isolate was better than the commercial isolate. Reduced solubility was probably a consequence of several factors, including: low net charge at neutral pH values of aggregates composed of partially degraded subunits, increased hydrophobicity and increased presence of both major proteins released from aggregates. Increased hydrophobicity arises from exposure of interior hydrophobic residues after hydrolysis of globulins. According to electrophoretic profiles, enzyme primarily acts on insoluble aggregates, thus freeing key proteins: legumin and vicilin. These major proteins differ both on hydrophilicity and on values of dissociation constants. Vicilin is a lot more hydrophilic than legumin. However, legumin is more prone toward dissociation and re-association. Both dissociation and re-association are pH dependable. At pH 7, legumin exists in its less soluble hexameric form and the presence of such a hexamer leads to a decrease in solubility. At pH <3 and pH >10, there is a tendency of dissociation of hexamers into monomers. At basic pH, dissociation occurs at a slower rate. Since at acidic pH, monomers are the most abundant form of legumin, their pronounced solubility dictates overall higher values of that parameter. At pH 8, legumin is most probably present in mixture of different forms, from hexamers to monomers. So, at this pH, solubility shows a tendency to rise, because of partial denaturation of legumin hexamer.

### 2.4. Emulsifying Properties

**Figure 4:**
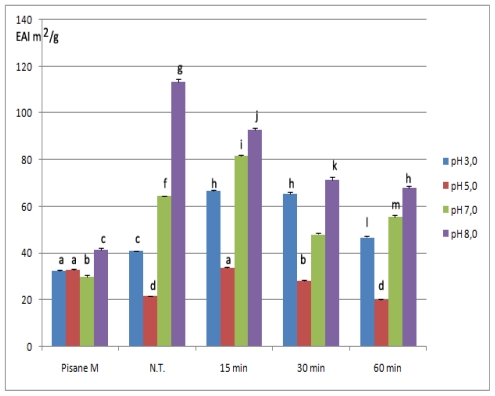
Emulsifying activity of commercial, non-modified (N.T.) and modified isolates *. * Bars with the same letter are not statistically different at *p* < 0.05. Means were of triplicate determinations.

**Figure 5:**
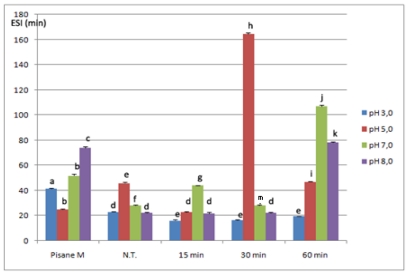
Emulsion stability of commercial, non-modified (N.T.) and modified isolates *. * Bars with the same letter were not statistically different at *p* < 0.05. Means were of triplicate determinations.

Emulsifying properties of native, commercial and modified isolates were expressed as emulsion activity index (EAI) and emulsion stability index (ESI) and are shown in Figures 4 and 5. EAI reflects the ability of the proteins to induce formation of the newly created dispersed particles in emulsions, whereas ESI value reflects their stability. Both parameters of non-modified isolates were pH-dependent with the minimum occurring near the isoelectric region (at pH 5.0; EAI 41.03 m^2^/g, ESI 23.03 min). Also, dependence between both parameters and pH had similar U-shape profile, which was in agreement with previous investigations based on PPI [41], and other plant proteins [42].

As could be seen, emulsifying properties, especially emulsifying capacity could be improved by chymosin. In addition, the effect of this enzyme was influenced by both duration of hydrolysis and pH at which emulsions were prepared.

Chymosin increased capacity of pea protein emulsions prepared at low pH (3.0 and 5.0). At pH 3.0, depending on duration of hydrolysis, EAI increased by 14.16–62.51% and ranged from 46.84 ± 0.5 m^2^/g to 66.68 ± 0.22 m^2^/g. In addition, EAI values samples hydrolyzed for 15 and 30 min were not statistically different (*p* < 0.05). Similarly, improved EAI values were observed at pH 5.0 using samples treated up to 30 min (28.05 m^2^/g), as well as with 15 min treated samples at pH 7.0 (81.64 m^2^/g). In contrast, at pH 8.0 emulsion capacity of modified samples was lower compared to initial isolates.

However, partial hydrolysis with chymosin had variable effect on emulsion stability. In general, with samples treated for 60 min emulsions were the most stable, but the maximum ESI value was detected with 30 min treated samples at pH 5.0 (164.29 min). At pH 3.0 modified isolates had slightly lower ESI values then initial isolates whereas improved stability of all samples was at pH 7.0. ESI of these samples at pH 7.0 increased 1.22 to 4.64 times.

According to results of this investigation, a moderate significant correlation (0.74, as shown in section 2.5) existed between solubility and EAI, while there was no significant correlation between solubility and ESI as well as between EAI and ESI. These correlations suggested that different factors affected these two parameters. Rise in emulsion capacity of modified isolates at pH 3.0, 5.0 could be attributed to improved solubility. Due to better solubility, products of hydrolysis were reached easier at the interface than non-hydrolyzed proteins and formation of a protective layer was enhanced. Lower capacity at pH 8.0 (although solubility was improved), indicated that other factors, such as flexibility and interactions between products of hydrolysis, might have played an essential role. On the other hand, it is well known that stability of emulsion depends on the strength of protein-protein interaction at the oil-water interface. It seemed that proteins of 15 and 30 min modified isolates (except 15 min treated samples at pH 3.0, and 30 min treated samples at 5.0) did not interact effectively to form as strong interfacial membranes as in the case of 60 min treated samples at higher pH values (7.0 and 8.0) and non-modified samples.

### 2.5. Foaming Properties

The property of proteins to form and to stabilize foams is important in the production of a variety of foods. Foams can be defined as a two-phase system consisting of air cells separated by a thin continuous liquid layer. As good foaming agents, proteins should form and stabilize foams rapidly and effectively at low concentration over the pH range which exists in various foods. Foaming properties of modified PPI were expressed as foam capacity (%FC) and foam stability (%FS) of 0.1% protein solutions. Obtained results are presented in Table 2. 0.1% solutions of non-modified isolates had good and pH dependent capacity, but low foam stability. Chymosin improved foam capacity of PPI at all pH values. Slightly lower capacity (by 15.15%) than non-modified PPI was detected only with samples treated 30 min when foam was prepared at pH 8.0. Foam capacity of other modified samples, depending on pH value, was in the range of 203.0% (30 min-treated samples at pH 3.0) to 354.5%. Maximum was obtained with 30 min treated samples at pH 5.0 and 60 min treated samples at pH 8.0.

According to our results, 15 min of chymosin action increased foam capacity at all pH values and depending on the pH, it was 293.4–339.4%. This means that after 15 min of hydrolysis samples with FC higher than initial PPI by 5.24–79.44% (Table 2) were produced. The most intensive rise was obtained at pH 5.0, *i.e*. Longer hydrolysis produced further increase at pH 5.0 (up to 30 min) and at pH 8.0 (up to 60 min), whereas at pH 3.0 and 7.0 these samples had lower capacity compared to 15 min treated isolates, but higher than initial isolate.

Chymosin had no positive effect on foam stability at pH 7.0 and 8.0. Foams formed at these values were extremely unstable and completely disappeared within 3 min. In contrast, at lower pH values, especially with isolates treated for 60 min, foam stability was improved. For example, stability of 60 min treated samples at pH 5.0 was about 6 times higher than initial isolate.

Correlation analysis (Table 3) showed that there was no significant relation between solubility and foam capacity, whereas negative moderate correlation (−0.60) was observed between solubility and foam stability. Also, there was negative moderate correlation (−0.77) between foam stability and EAI which could serve as an indication that different factors influenced these parameters.

From these facts it could be deduced that various factors might have contributed to quicker foam formation combined with its stability in modified samples. It is known that a rise in both diffusion speed of protein to interface and speed of denaturation on interface causes enhancement in ability of sample to form foams. These values, in turn, depend on the molecular mass, surface hydrophobicity, and the stability of the conformation. Lower FC values of modified isolates compared to PPI can generally, be regarded as a consequence of smaller molecular masses of hydrolysis products. Difference in molecular masses of products of hydrolysis of major proteins might be explained by difference in their characteristics, such as: tendency toward dissociation, flexibility and ratio of hydrophilic to hydrophobic parameters. The stability of foam depends on the strength of the protein film and its permeability for gases. Pronounced instability of foams at higher pH values is probably due to the fact that, at this pH, both vicilin and convicilin are in their trimeric forms, while legumin is in the form of a hexamer. Because of high molecular masses, these proteins cannot form strong bonds at air/water interface, thus they experience a lot more gravitational pull than un-modified isolates.

The results obtained in this work suggested that chymosin can be a useful agent for improving emulsifying and foaming properties of pea proteins, especially at low pH values. In order to achieve better functionality of hydrolizates in a wide range of pH, it can be very useful to combine them with other components such as mono-and disaccharides. According to Herceg *et al.* [43], addition of carbohydrates in solutions of whey proteins resulted in significant improvement of foaming and emulsifying properties. Since serum milk proteins and pea proteins differ significantly in their properties, the effect of added sugars on emulsifying and foaming properties of chymosin-modified pea isolates must be further investigated.

## 3. Material and Methods

### 3.1. Isolate Modification

Pea protein isolate was prepared by isoelectric precipitation, according to method previously described by Barac *et al.* [29]. Initial material was dried peas of experimental line L1 (selected by the Institute of Field and Vegetable Crops, Smederevska Palanka, Serbia). Uniform suspensions of initial isolate were obtained by dispersing 5 g of lyophilized isolate in 100 mL of miliQ water and stir for 15 min. This step was done in triplicate. To avoid isoelectric precipitation of dominant pea proteins at pH 5.0 (which is the optimum pH value for chymosin activity), isolates were modified at pH 6.8 which corresponds to pH of raw milk. At this pH values, we ensured good solubility of proteins and their availability to enzyme. pH of the obtained suspensions was adjusted to 6.8 with 1 M HCl and incubated for 15 min at 37 °C. Dispersions were individually hydrolyzed with 25 mg of recombined chymosin (Maxiren, DSM, Denmark) for either 15 or 30 or 60 min, with constant stirring. The enzyme to substrate (E/S) ratio was 1:200. During hydrolysis, pH of reaction mixtures was periodically (every 5 min) adjusted to pH 6.8. pH of resulting hydrolysates was adjusted to 7.0, then samples were heated at 60 °C for 3 min to inactivate the enzyme and immediately cooled in ice bath, lyophilized, ground and stored.

### 3.2. Degree of Hydrolysis

Degree of hydrolysis (DH) was determined according to Kim *et al.* [24]. Both amount of soluble nitrogen and amount of total nitrogen was evaluated using method of Helrich [44]. Amount of soluble nitrogen was measured in supernatant obtained by mixing by 10 mL of inactivated hydrolysate with 10 mL of 20% TCA and centrifuging at 12,000 g for 15 min. Total nitrogen was determined from 10 mL of suspension prepared in the same way as for enzymatic hydrolysis, but without the enzyme. DH was calculated using amount of soluble proportion *vs.* the amount of total nitrogen in protein isolate suspension.

### 3.3. Native-PAGE

Protein hydrolysates were analyzed by PAGE, following the general procedure by Davis [45] using 7% (w/v) acrylamide gels. 2 mg of lyophilized hydrolyzates were dissolved in 1 mL of 0.03 M Tris-HCl buffer containing 0.01 M 2-mercaptoethanol (pH 8.0). A 25 μL sample was loaded per well. The gels were run at 30 mA per gel for 5 h to completion. Gels were fixed, stained with 0.1% (w/v) Coomassie Blue R-250 (dissolved in 12% (v/v) acetic acid, and 50% (v/v) methanol) for 45 min and de-stained with 7% (v/v) acetic acid and 5% (v/v) methanol for 48h.

### 3.4. SDS-PAGE

SDS-PAGE was conducted following the procedure by Fling and Gregerson [46] using 5% (w/v) stacking and 12.5% (w/v) resolving gel. Prior to electrophoresis, 2 mg of protein hydrolysates was dissolved in sample buffer (0.055 M Tris-HCl, pH 6.8, 2% (w/v) SDS, 7% (v/v) glycerol, 4.3% (v/v) β-mercaptoethanol, 0.0025% (w/v) bromophenol blue), heated at 90 °C for 5 min and cooled at the room temperature. 25 μL of non-treated samples and 50 μL of modified samples was loaded per well. The gels were run at 30 mA per gel for 6 h to completion. Gels were fixed, stained with 0.23% (w/v) Coomassie Blue R-250 (dissolved in 3.9% (w/v) trichloroacetic acid (TCA), 6% (v/v) acetic acid, and 17% (v/v) methanol) for 45 min and destained with 8% acetic acid and 18% (v/v) ethanol. Molecular weights of the polypeptides were estimated by using low molecular weight calibration kit (Pharmacia, Sweden). Molecular weight markers included: phosphorylase B (94.0 kDa), bovine albumin (67.0 kDa), ovalbumin (43.0 kDa), carbonic anhydrase (30.0 kDa), soybean trypsin inhibitor (20.1 kDa), and α-lactalbumin (14.4 kDa).

PAGE and SDS-electrophoresis of proteins were performed in duplicate by placing two aliquots of same sample on two gels that were run simultaneously in same electrophoretic cell.

### 3.5. Densytometric Analysis

SDS-gels were scanned and analyzed by SigmaGel software version 1.1 (Jandel Scientific, San Rafalel, CA). The determination of vicilin and legumin was made, and their concentrations and ratio were calculated from the sum of the total area of their subunits. Each pattern was analyzed in triplicate.

### 3.6. Protein Solubility

Protein solubility was determined according to the method of Wu *et al.* [18]. Four samples of different pH (3.0; 5.0; 7.0 and 8.0) were prepared by following: 20 mg of each sample were dispersed in 20 mL milliQ water and stirred until uniform dispersions were formed (30 min). pH of each suspension was then adjusted to desired value with 1 M NaOH or 1M HCl. Suspensions were then stirred for 1 h and supernatant was produced by centrifuging for 15 min at 17,000× g (Sigma, Germany). In supernatant protein content was measured using method of Bradford [47]. To determine total protein content, 20 mg of isolate was extracted with 20 mL of 0.5 M NaOH. Protein solubility was then calculated by following equation:

Solubility (%)=protein content in supernatant/total protein content×100

As a control for solubility, as well as for other investigated properties we used commercial pea protein isolate Pisane M (Cosucra, Belgium).

### 3.7. Emulsifying Properties

Emulsifying properties were measured according to procedure of Pearce and Kinsella [48]. Emulsions were prepared by homogenizing 45 mL 0.1% protein suspension of isolate with 15 mL of pure sunflower oil in mechanical homogenizer for 1 min at the highest settings. Samples (500 μL) were taken at 0 min and 10 min after homogenization from the bottom of the container. Both samples were diluted with 10 mL 0.1% SDS solution. Absorbances of these diluted emulsions were measured at 500 nm and values for absorbances at 0 min (A_0_) and 10 min (A_10_) after emulsion formation were recorded. Emulsifying activity index (EAI) and emulsifying stability index (ESI) were calculated using these values. EAI and ESI were measured in two different days. Each day two different emulsions of the same sample were produced and three aliquots of each emulsion were taken for further analysis.

### 3.8. Foaming Properties

Foaming properties were expressed as foaming capacity (FC) and foam stability (FS) as described by Barac *et al.* [29]. Foaming was attained by bubbling a stream of air (6 dm^3^·min^−1^) during 15 s through a Waters filter holder (Waters, USA) placed at the bottom of a 250 mL graduated column containing 30 mL 0.1% protein solution in water adjusted to pH 3.0, 5.0, 7.0 and 8.0. Total volume was taken at 0 min for foam capacity and at 3 min for foam stability. Foaming properties were expressed as:

$$\begin{array}{c} \text{FC}(\%)=\text{volume after bubbling}-\text{volume before}\;\text{bubbling}/\text{volume before bubling}\times 100\\ \text{FS}(\%)=\text{residual volume after }3\;\text{min}/\text{total foam volume}\times 100 \end{array}$$

### 3.9. Statistical Analysis

The data were analyzed using Statistica software version 5.0 (StatSoft Co., Tulsa, OK). Significant difference between mean values was determined by *t*-test procedure for independent samples. Also, regression analyses were carried out.

## 4. Conclusions

According to our data, recombined chymosin caused a low degree of hydrolysis (3.9–4.7%). Yet, functional properties of PPI were improved, especially at low pH values (3–5). At these pH values, solubility, emulsifying capacity and foaming stability of all isolates were higher than the initial ones. Longer-treated samples (60 min) formed more stable emulsions at higher pH values (7.0, 8.0) compared to non-treated isolates. Also, regardless of pH value, foaming ability was improved for all treated isolates. A moderate positive correlation between solubility and emulsifying capacity (0.74) and negative correlation between solubility and foam stability (−0.60), as well as between FS and EAI (−0.77), were registered. Described improvements of functional properties were the result of partial hydrolysis of insoluble protein complexes.

## Figures and Tables

**Table 1:** The ratio of major proteins in non-treated and modified isolates *.

		Modified isolates		

	Non-treated	15 min	30 min	60 min
Vicilin	37.88 ^a^	33.52 ^b^	34.57 ^c^	34.3 ^c^
Legumin	32.96 ^a^	23.6 ^b^	26.15 ^c^	24.43 ^d^
V/L **	1.15 ^a^	1.42 ^b^	1.32 ^c^	1.40 ^d^
V+C/L ***	1.46 ^a^	1.87 ^b^	1.74 ^c^	1.92 ^d^

* Means in the same row marked with the same letter were not statistically significant at *p* < 0.05,

** vicilin to legumin ratio,

*** sum of vicilin and convicilin to legumin ratio.

**Table 2:** Foaming properties of non-modified and modified isolates *.

Foaming properties (%)										

pH	Pisane M		N.T.		Modified isolates					

					15 min		30 min		60 min	

	FC	FS	FC	FS	FC	FS	FC	FS	FC	FS
3.0	324.2 ± 3.30 ^a^	89.6 ± 0.04 ^a^	187.9 ± 3.30 ^b^	27.4 ± 0.03 ^b^	293.4 ± 3.10 ^c^	17.56 ± 0.60 ^b^	203.0 ± 0.60 ^d^	10.45 ^c^	263.6 ± 1.70 ^e^	48.27 ± 0.50 ^d^
5.0	339.4 ± 4.40 ^a^	6.3 ± 0.03 ^a^	172.7 ± 8.40 ^b^	12.3 ± 0.04 ^b^	309.9 ± 4.10 ^c^	11.74 ± 1.16 ^b^	354.5 ± 6.50 ^d^	35.90 ^c^	278.8 ± 3.40 ^e^	72.82 ± 0.20 ^d^
7.0	270.8 ± 4.20 ^a^	0 ^a^	203.0 ± 4.20 ^b^	0 ^a^	339.4 ± 6.50 ^c^	0 ^a^	278.8 ± 8.10 ^a^	7.61^b^	278.8 ± 3.00 ^a^	0 ^a^
8.0	203.0 ± 4.70 ^a^	0 ^a^	278.8 ± 6.40 ^b^	45.6 ± 0.03 ^b^	293.4 ± 4.20 ^c^	0 ^a^	263.6 ± 5.60 ^d^	0 ^a^	354.5 ± 4.20 ^e^	0 ^a^

* Values marked with the same letter within same row and same parameter were not different, N.T. nontreated isolate.

**Table 3:** Correlation coefficients between functional properties of pea protein isolates *.

	Foam capacity (%)	Foam stability (%)	EAI	ESI
Solubility (%)	−0.39	−0.60 *	0.74 *	−0.33
Foam capacity (%)		−0.01	−0.05	0.56
Foam stability (%)			−0.77 *	0.20

* Values marked with ^*^ were statistically significant.
